# Few-cycle localized plasmon oscillations

**DOI:** 10.1038/s41598-020-69761-x

**Published:** 2020-07-31

**Authors:** Mária Csete, András Szenes, Dávid Vass, Balázs Bánhelyi, Péter Dombi

**Affiliations:** 1 Department of Optics and Quantum Electronics, University of Szeged, 6720 Szeged, Hungary; 2 Department of Computational Optimization, University of Szeged, 6720 Szeged, Hungary; 3 Wigner Research Centre for Physics, 1120 Budapest, Hungary; 4 ELI-ALPS Research Institute, 6728 Szeged, Hungary

**Keywords:** Nanoparticles, Nanophotonics and plasmonics, Ultrafast photonics

## Abstract

The generation of few-cycle laser pulses proved to be a key enabling technology in strong-field physics and ultrafast science. The question naturally arises whether one can induce few-cycle localized plasmon oscillations in optical near-fields. Here, we perform a comparative study of different plasmonic nanoresonators illuminated by few-cycle pulses. We analyze the number of cycles (NOC) of the plasmonic field, the near-field enhancement (NFE) as well as the figure of merit NFE/NOC. The pulse length dependence of these quantities is also investigated. Throughout the inspected pulse-length interval silica-gold and silica-silver core–shell monomers have the potential to preserve the NOC of the incoming pulse, silver bow-ties result in the highest NFE, whereas gold core–shell dimers have the highest NFE/NOC. Based on the analysis, silver bow-ties, gold core–shell and silver nanorod dimers proved to be the most suitable for few-cycle near-field amplification.

## Introduction

Few-cycle laser pulses (especially with carrier-envelope phase stabilization) proved to be highly important in many experiments in strong-field physics, attosecond science and pump-probe methods with ultrahigh time resolution. Therefore, the question naturally arises whether one can combine extreme temporal concentration of electromagnetic energy (in the form of few-cycle pulses^1,2^) with ultrahigh spatial localization of laser fields readily provided by localized surface plasmon (LSP) resonance. Here, we investigate the possibility of the generation of few-cycle LSPs by analyzing the temporal response of different types of plasmonic nanoparticles and nanoparticle dimers.

Plasmonic metal nanorods have already been investigated extensively. They inherently show plasmonic resonances corresponding to their short and long axes. The transversal and longitudinal resonance frequencies depend on the material, axis lengths and aspect ratio of the nanorod. Hence, nanorod LSP resonance can be tuned through wide spectral intervals by changing the geometry^3,4^.

Dielectric-metal core–shell type nanoresonators offer unique possibility of far-field and near-field control spanning wide spectral intervals via tailoring the plasmon hybridization promoted by two metal-dielectric interfaces^5,6^. An interesting possibility is that dipolar resonance can be achieved at the same wavelength in two different generalized aspect ratio (*GAR* = *R*_inner_/*R*_outer_) intervals by applying the same core and shell materials^7^. The thick (thin) shell composition supports a plasmonic resonance, which is accompanied by a spectrally wide (narrow) scattering cross-section maximum and low (high) absorbance. Although the scattering of core–shell nanoparticles is always lower than that of a homogeneous sphere, the achievable broad bandwidth and the possibility to control the Q factor of the plasmonic resonance by varying the GAR offers the unique possibility of electric field enhancement combined with the preservation of few-cycle transients^8,9^.

More complex plasmonic structures were also proposed and then used to realize plasmon enhanced spectroscopy^10^. The achievable electric field enhancement and temporal evolution of the optical response were investigated on monomer and dimer Ag and Au nanoparticles as well as on silver-gold core–shell bimetal nanoparticles^11^. It was shown that the electric field enhancement on them is higher than on metal spheres composed of either metals, however the plasmonic decay dynamics is slightly different on alloy core-shells.

Bow-tie structures are very efficient plasmonic dimers that can be fabricated via lithography on substrates hence they are frequently used in applications. Further advantage of bow-tie structures is their low apex curvature, which makes it possible to reach high near-field enhancement (NFE) in the nanogap^12–14^.

Accordingly, for few-cycle plasmonic field generation, various plasmonic dimers are promising candidates, which offer higher electric field confinement and more degrees of freedom in time-evolution control^15–18^. Solid sphere dimers were shown to be applicable as coherent, highly localized and tunable photon sources^18^. In their junction, the relative strength of second-order nonlinearities, such as second harmonic and sum frequency generation as well as third-order nonlinearities, such as four-wave mixing, can be controlled by varying the dimer symmetry. For higher photon energies, frequency doubling occurs and two-photon luminescence emerges, governed by the bandstructure of bulk metals which is typically gold in applications. Intense third harmonic generation and emission occurs from the volume close to dimer gaps, which makes it possible to probe nanoscale optical properties such as sub-cycle plasmon dephasing time, which is in the order of 2 fs^19^. In case of point symmetry, the second-order nonlinearities are suppressed, while four-wave mixing remains unaffected in the visible frequency range, moreover its intensity can be modulated by tuning the dimer separation. Such a nanoscale photon source can be applied as a building block of integrated plasmonic devices and as a near-field optical probe^20^.

The interaction of localized plasmonic modes and propagating waveguide modes in a substrate were shown to result in plasmon dephasing time modification, proving the role of array effects in pulse shaping^21^. Strong-field photoelectron emission from plasmonic nanostructures was demonstrated to show a well-defined correlation with the plasmonic resonance of nanoparticles^22,23^. The photoemission and electron re-scattering effect proved to be useful to measure the plasmonic field enhancement factor precisely within a less than 1 nm layer from the nanoparticles surface^24^. However, several studies revealed that melting prevents reaching higher near-field intensities in the gaps of dimers^25^. In addition, few-cycle near-fields at simple nanotips brought important discoveries in photoemission experiments^26–28^. Possible applications are also in the next generation of photonic circuitries. Ultrafast, femtosecond optical switching and terahertz modulation bandwidth are important tools in all-optical signal transfer, which has the potential for orders of magnitude faster operation speed than that of already existing technologies^29^. By using few-cycle plasmonic fields SERS techniques can be extended to the ultrafast regime, where direct probing of plasmon mediated chemical processes including bond-making and breaking down to femto- and picosecond timescale becomes possible^30^. Single-cycle LSP generation is accompanied by extreme concentration of the energy both in temporal and spatial domains, which makes it possible to study strong-field phenomena already at low laser energy^31^. Surface plasmons can also be exploited in high harmonic generation required to achieve extreme ultraviolet pulses^32^ and in several novel application areas of strong-field nano-optics in the ultrafast regime^33^.

The present work focuses on the general question, which type of plasmonic nanoresonator is capable of producing significant near-field enhancement, while preserving the few-cycle nature of the near-field response. Accordingly, several plasmonic nanoresonator types, namely single nanorods and core–shell particles, nanorod and core–shell dimers as well as bow-ties have been thoroughly inspected and an optimization method was developed yielding the best geometries for these purposes.

## Methods

The concept of this research is that the few-cycle characteristic of the plasmonic field can be ensured via nanoresonators, which possess broad spectral responses. The plasmonic resonance is accompanied by almost coincident absorption (ACS) and scattering cross-section (SCS) maxima in case of nanorods and bow-ties, whereas the core-shells exhibit a broad SCS peak with a detuned ACS maximum^7^. Accordingly, the desired resonant behavior can be ensured uniformly by tuning the scattering cross-section (SCS) maximum of the plasmonic nano-objects to the target wavelength (800 nm, for our case). The coincident ACS maximum allows to maximize the near-field enhancement in case of nanorods and bow-ties. In case of core–shell nanoresonators a trade-off arises, since usually a good-quality resonator possessing a narrow scattering cross-section makes it possible to achieve strong electric fields around the plasmonic objects, rather than a loss caused by the resistive heating of the nanoparticle. By applying pre-designed plasmonic architectures, which ensure LSP resonance tunable throughout a wide spectral interval accompanied by a properly large scattering cross-section bandwidth, the quality factor of the resonance can be controlled, and as a result, the few-cycle characteristics of the illuminating pulse can be preserved in the enhanced near-field response.

A finite difference time-domain method (Lumerical FDTD Solutions) was applied to include the time-parameter into the modeling of electromagnetic near-field distribution around various plasmonic nanostructures. Monomer and dimer nanoresonators were placed on silica-glass substrate-air interface and illuminated by few-cycle laser pulses in total-field scattered-field configuration. The mesh was locally improved around the nanoresonators, the smallest element was 0.5 nm in the dimers’ nanogap, whereas in the dielectric media the mesh was *λ*/5 scaled. Tabulated data sets were used to include the wavelength dependent dielectric properties of metallic materials^34^. Conditional geometry optimizations were realized to ensure full control over the characteristics of the response of nanoresonators by using an in-house developed algorithm^35^.

The goal was to obtain the maximum near-field enhancement in proximity of the inspected nanoresonators at 800 nm central wavelength of the excitation pulse. Additional optimization criteria were set to ensure a resonant behavior and to preserve the few-cycle response simultaneously. Namely, a primary criterion regarding that the wavelength of the SCS maximum accompanying the plasmonic resonance has to be near 800 nm was set. In addition to this, a variable measuring the number of optical cycles (NOC) in the near-field within the full-width-at-half-maximum (FWHM) of the intensity envelope was defined. Its nanoresonator type dependent value was required to be lower than 2.5 for core–shell monomers and dimers, whereas for nanorod monomers, dimers and bow-ties this value had to be less than 1.7. The geometrical parameters were varied in the following intervals: (i) the long and short axis of nanorod monomers *a*_x_: [20 nm, 400 nm] and *a*_y_: [20 nm, 200 nm]; the inner and outer radius of core–shell monomers: *R*_inner_: [50 nm, 200 nm] and *R*_outer_ = *R* + *t*: [55, 300], where the minimal *t* shell thickness was 5 nm. The vertical edge (*L*), horizontal extension (*W*), the corresponding, opening angle (α) and thickness (*T*) of the bow-tie composing triangular antennas were *L*: [50 nm, 250 nm], W: [50 nm, 250 nm], α: [45°, 135°], *T*: [20 nm, 100 nm]. The same nanoresonator parameter intervals were applied for nanorod and core–shell dimers, as for their monomer counterparts. The core–shell dimers were inspected primarily, since in thick-shell (small *GAR*) composition the wide SCS necessary for minimal NOC is compromised with a weaker LSP resonance accompanied by smaller absorptance and NFE. The *d* distance was varied in the [4, 25 nm] interval in case of core–shell dimers, whereas the optimal core–shell distance was transmitted to nanorod dimers and bow-ties, in order to ensure the dimer’s comparability.

The optimizations were performed by using linearly polarized Gaussian light pulses of 2.7 fs length; the NOC of the excitation pulse is 1.1 in this case. Subsequently, the wavelength dependence of the scattering cross-section was determined via numerical computations for all inspected structures. This was realized for all inspected pulse lengths to enable comparison with the spectral distribution of each pulse that we used. The time-dependent response of the optimized nanoresonators as a function of pulse length was also investigated, namely all optimized nanoresonators were re-illuminated by 4 and 5 fs pulses as well, corresponding to 1.56 and 1.92 optical cycles. The NOC and NFE were determined based on the E_x_ component parallel to the **E**-field oscillation direction of the incoming pulse that was monitored at 1 nm distance uniformly from the nano-objects.

The fundamental studies were performed on individual nanorods and core–shell particles, nanorod and core–shell dimers as well as on bow-ties. To realize a comparative study, the time-evolution of the optical response and the achieved electric field enhancement were compared for the optimized nanoresonators. The pulse length dependence of NOC, NFE and the figure-of-merit FOM = NFE/NOC was also studied.

## Results and discussion

The SCS in all optimized configurations exhibits one single broadband peak inside the 800 ± 20 nm wavelength range according to the broadband resonance of optimized configurations. It is also a common characteristic that the scattering cross-section spectra are almost independent of the length of the incoming pulse in the inspected wavelength interval, due to the sufficiently large spectral overlap. All optimized nanoresonators exhibit the typical time-response of near-resonant nanoantennae so that after reaching a maximum, a nearly exponential smooth decay is observed. The longer (shorter) decay compared to free space correlates with the narrower (broader) SCS spectrum of the resonant plasmonic nanoresonators. Considerable NFE is achieved with all inspected resonators, therefore the free-space pulses were rescaled to ensure comparison of the time evolutions. The NFE of optimized structures for different pulse lengths is almost identical, only a slight decrease is observable by increasing the pulse length. The achieved optical response parameters are summarized in Table 1, with more details given in the subsequent sections.

**Table 1:** Optical response of optimized nanoresonators.

	Pulse-length	Au rod monomer	Ag rod monomer	Au core–shell monomer	Ag core–shell monomer	Au rod dimer	Ag rod dimer	Au core–shell dimer	Ag core–shell dimer	Au bow-tie	Ag bow-tie	free space
NOC	2.7 fs	1.6	1.7	1.07	1.07	1.5	1.49	1.17	1.16	1.63	1.53	1.1
	4 fs	2.05	2.15	1.54	1.54	1.95	1.94	1.63	1.61	2.06	1.96	1.56
	5 fs	2.39	2.49	1.9	1.9	2.3	2.28	1.98	1.96	2.41	2.31	1.92
NFE	2.7 fs	9.06	12.45	2.99	2.93	39.32	41.37	37.58	22.92	42.33	42.87	1
	4 fs	9.06	12.49	2.98	2.93	39.24	41.29	37.54	22.89	42.33	42.87	1
	5 fs	9.05	12.42	2.98	2.93	39.22	41.26	37.52	22.87	42.3	42.52	1
NFE/NOC	2.7 fs	5.66	7.32	2.79	2.74	26.2	27.7	32.12	19.76	25.97	28.02	0.91
	4 fs	4.42	5.81	1.94	1.9	20.18	21.33	23.03	14.22	20.55	21.87	0.64
	5 fs	3.79	4.99	1.57	1.54	17.08	18.08	18.95	11.67	17.55	18.41	0.52
NOC slope (fs^−1^)		0.34	0.34	0.36	0.36	0.35	0.34	0.35	0.35	0.34	0.34	0.36
NFE slope (fs^−1^)		0	− 0.01	0	0	− 0.04	− 0.05	− 0.02	− 0.01	− 0.01	− 0.14	0
NFE/NOC slope (fs− ^1^)		− 0.82	− 1.02	− 0.54	− 0.53	− 3.4	− 4.22	− 5.79	− 3.55	− 3.69	− 4.2	− 0.17

### Nanorod monomers

**Figure 1:**
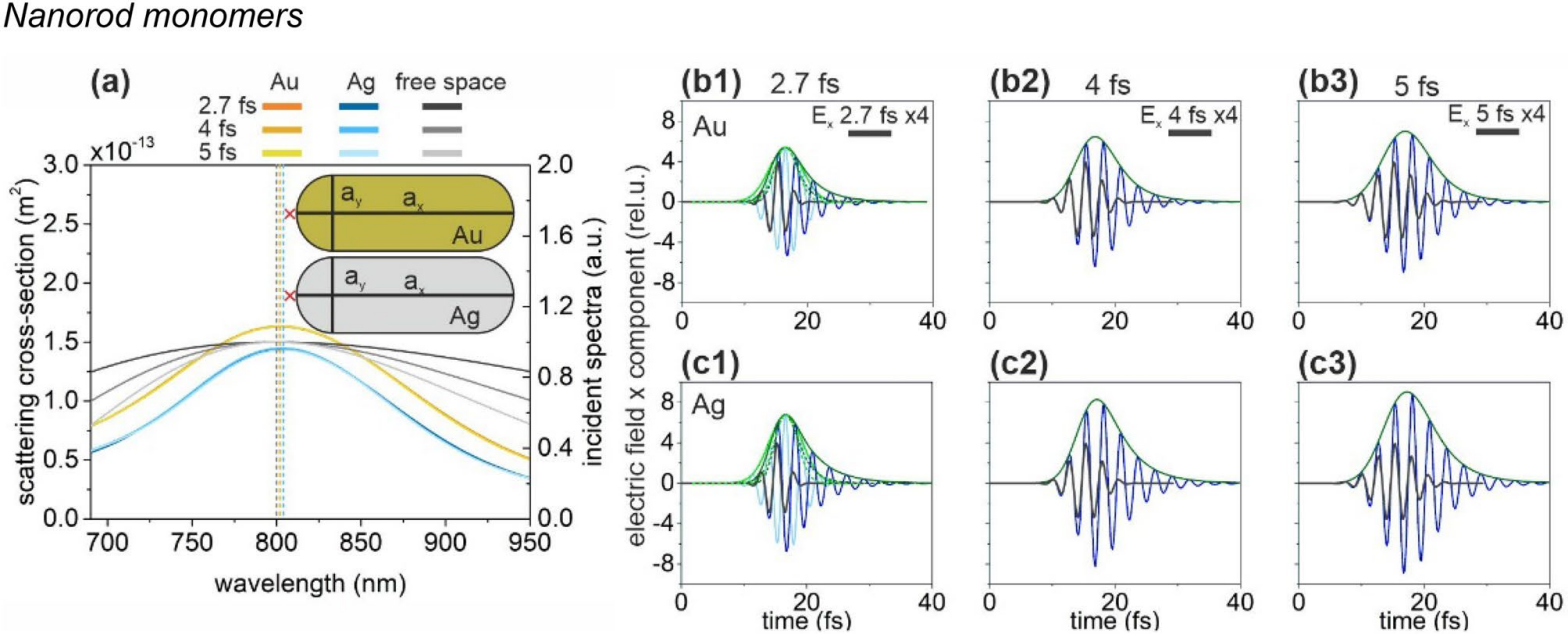
(**a**) Scattering cross-section of optimized nanorod monomers and the spectral distribution in free-space in case of illumination by 2.7-fs, 4-fs and 5-fs excitation pulses. Insets: Geometry and tuned parameters of nanorod monomers. The red cross indicates the location of the near-field read-out. (Optimal parameters: a_x_ = 217 nm, a_y_ = 82 nm for Au nanorod and a_x_ = 203 nm, a_y_ = 62 nm for Ag nanorod). Temporal decay of the enhanced plasmonic field in the proximity of (**b**) the gold and (**c**) the silver nanorod monomer; E_x_: 2.7 fs excitation (black), 1.7 NOC criterion (light blue), plasmonic (blue), E_x_ (intensity) envelope: 1.7 NOC criterion solid (dashed) green, plasmonic solid (dashed) olive.

The geometry and SCS of optimized Au and Ag nanorod configurations are similar (Fig. 1a). Although, their resonance peaks are close to 800 nm, the FWHM of both SCS peaks is slightly lower than that of the spectrum corresponding to the exciting 2.7 fs pulse, which causes a slightly longer plasmonic field decay (Fig. 1b,c, Table 1). Namely, both nanorods enhance the incoming pulse intensity, however after reaching the electric field maximum, a slightly longer exponential decay is observable than for the profile of the incoming pulse.

The broader SCS of the optimized gold nanorod results in slightly lower NOC (1.60) in the intensity envelope than that in case of the silver nanorod (1.70). The SCS maximum is slightly lower according to the smaller Ag nanorod volume, which promotes higher, 12.45-fold NFE. The NFE/NOC ratio is considerably higher in case of the silver nanorod for each inspected pulse length, according to the higher NFE, which makes Ag nanorods better candidates for few-cycle pulse enhancement (Table 1).

### Core–shell monomers

**Figure 2:**
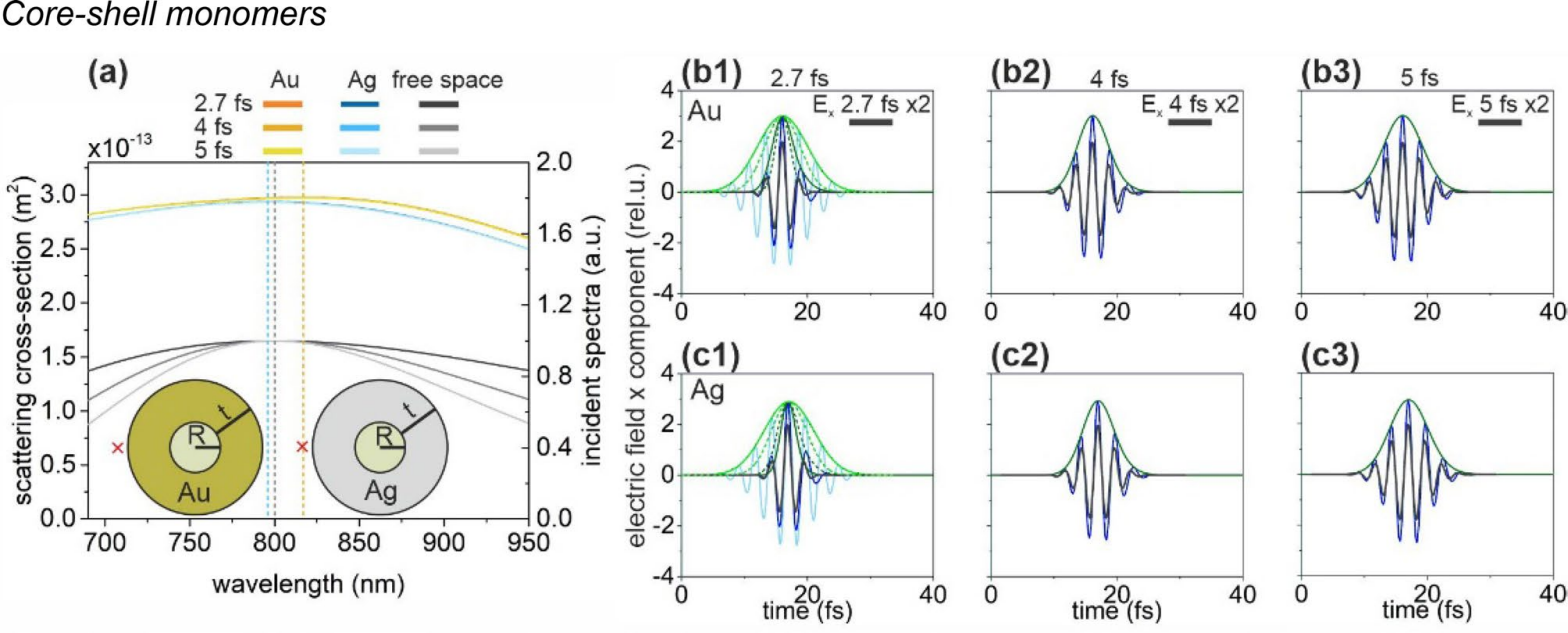
(**a**) Scattering cross-section of optimized core–shell monomers and the spectral distribution in free-space in case of illumination by 2.7-fs, 4-fs and 5-fs excitation pulses. Insets: Geometry and tuned parameters of core–shell monomers. Red cross indicates the location of the near-field read-out. (Optimal parameters: R = 69 nm, t = 80 nm for silica-Au core–shell monomer and R = 67 nm, t = 80 nm for silica-Ag core–shell monomer). Temporal decay of enhanced plasmonic field in the proximity of (**b**) the silica-gold and (**c**) the silica-silver core–shell monomer; E_x_: 2.7 fs excitation (black), 2.5 NOC criterion (light blue), plasmonic (blue), E_x_ (intensity) envelope: 2.5 NOC criterion solid (dashed) green, plasmonic solid (dashed) olive.

Analogously to nanorods, the SCSs of optimized silica-gold and silica-silver core–shell nanospheres are similar (Fig. 2a). These nanoresonators show an ultrabroadband plasmonic resonance, the peak of which is broader than that of the incoming 2.7 fs pulse. Although, both core–shell monomers result in a considerable intensity enhancement, the NFE is the smallest among all inspected monomers in accordance with their resonant behavior as a thick-shell type concave nanoresonator (Fig. 2b,c, Table 1).

The SCS peak of the optimized silica-gold core–shell nanostructure is slightly higher and broader, however the NOC of the nanostructures with different shell materials are identical, with values of 1.07, 1.54 and 1.90 for 2.7-fs, 4-fs and 5-fs input pulses, respectively. The slightly smaller NOC with respect to the incoming pulse indicates slightly longer apparent wavelength in the total near-field independently of the pulse length. The slightly higher SCS correlates with the slightly larger size of the silica-gold core–shell. Surprisingly, both Au and Ag coated core-shells show approximately threefold NFE at 2.7 fs, however the NFE is slightly lower in the vicinity of the optimized silica-silver despite the significantly lower achieved detuning and inherent advantages of this material in plasmonic field enhancement. The larger NFE of the silica-gold core–shell correlates with the presence of a local maximum on the absorption cross-section at the central wavelength, which indicates a more well defined LSP.

According to the similar NFE and same NOC values, the NFE/NOC ratios are close to each other in case of silica-gold and silica-silver core–shell monomers, however the NFE/NOC is slightly higher in presence of gold coating for each inspected pulse length, in accordance with the larger NFE (Table 1).

Due to the significantly broader spectra 1.5-times lower NOC is reachable with the optimized core–shell structure compared to nanorods. This indicates that the core–shell monomers are better suitable to preserve few-cycle characteristics (Table 1). In contrast, optimized Au (Ag) coated core–shell nanoresonators generate 3 (4)-times lower NFE, resulting in ~ 2 (~ 3)-times lower NFE/NOC ratio, respectively. The latter is caused by the trade-off between the achievable NOC and NFE and indicates that among singlet nanoresonators, the core–shell type is a weaker candidate, when high near-field enhancement is also required.

### Nanorod dimers

**Figure 3:**
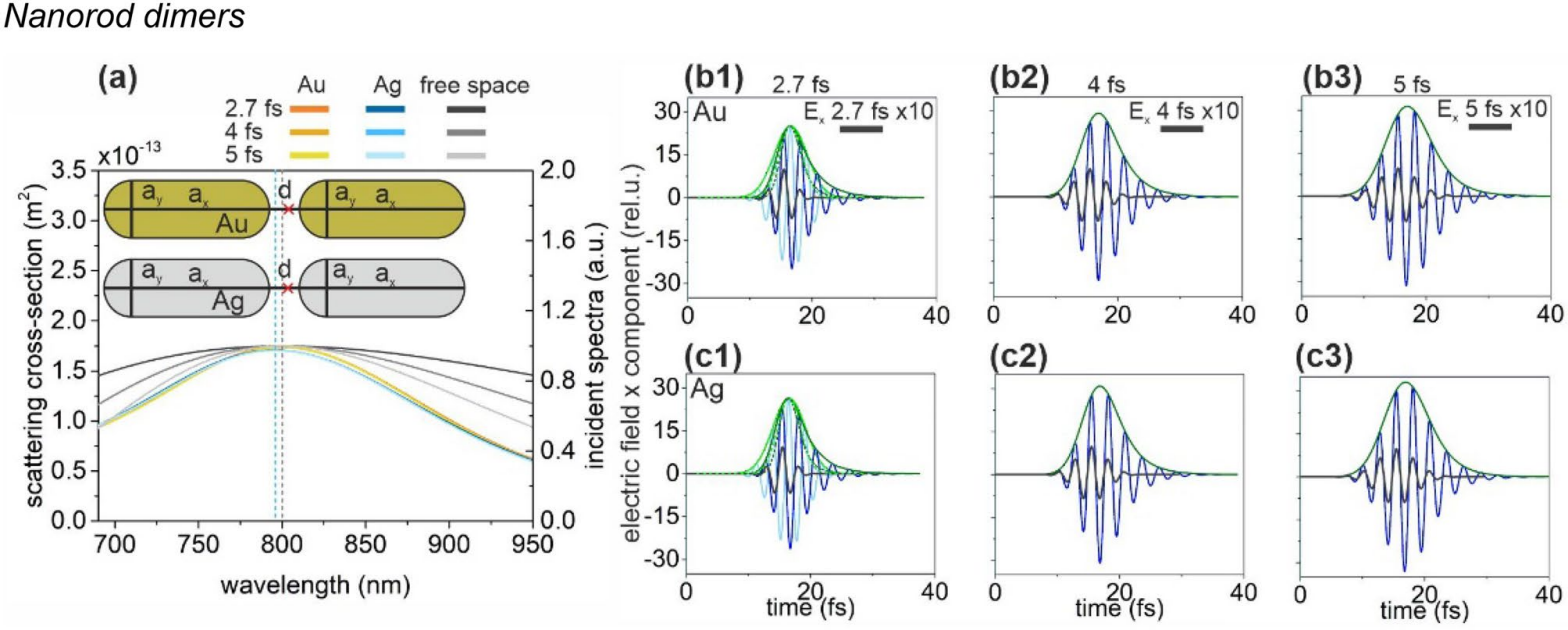
(**a**) Scattering cross-section of optimized rod dimers and the spectral distribution in free-space in case of illumination by 2.7-fs, 4-fs and 5-fs excitation pulses. Insets: Geometry and tuned parameters of nanorod dimers. Red cross indicates the location of the near-field read-out. (Optimal parameters: a_x_ = 164 nm, a_y_ = 68 nm, d = 10 nm for Au nanorod dimer and a_x_ = 156 nm, a_y_ = 60 nm, d = 10 nm for Ag nanorod dimer). Temporal decay of the enhanced plasmonic field in the gap of (**b**) the gold and (**c**) the silver nanorod dimer; E_x_: 2.7 fs excitation (black), 1.7 NOC criterion (light blue), plasmonic (blue), E_x_ (intensity) envelope: 1.7 NOC criterion solid (dashed) green, plasmonic solid (dashed) olive.

Optimized gold and silver nanorod dimers have narrower SCS spectra than the spectrum of the incoming 2.7-fs pulse (Fig. 3a). Both SCS spectra are well centered at around 800 nm.

Because of their narrower spectrum, both the gold nanorod dimer and the silver nanorod dimer exhibit a significantly longer plasmonic decay than the duration of the input pulse (Fig. 3b,c, Table 1). The SCS peaks of the optimized gold and silver nanorod dimer exhibit similar FWHM, hence the achieved 1.50 and 1.49 NOC values at 2.7 fs input are similarly higher than the NOC of the incoming pulse (1.10). Both nanorod dimers exhibit higher NOC than that of the input pulse in case of longer pulses, as well (Table 1). The NFE is considerably higher in case of silver nanorod dimers independently of the pulse length.

According to the opposite behavior of NFE and NOC values, the NFE/NOC ratio is slightly less balanced, and as a result it is slightly higher in case of silver nanorod dimers independently of the pulse length.

Changing from nanorod monomers to dimers is advantageous for a lower NOC value, which is smaller independently of material composition (Table 1). The NFE of both nanorod dimers is also significantly higher than that of the corresponding monomers, namely ~ 4 (3)-times higher NFE is achieved via Au (Ag) nanorod dimers, respectively. This results in significantly higher NFE/NOC in case of nanorod dimers.

### Core–shell dimers

**Figure 4:**
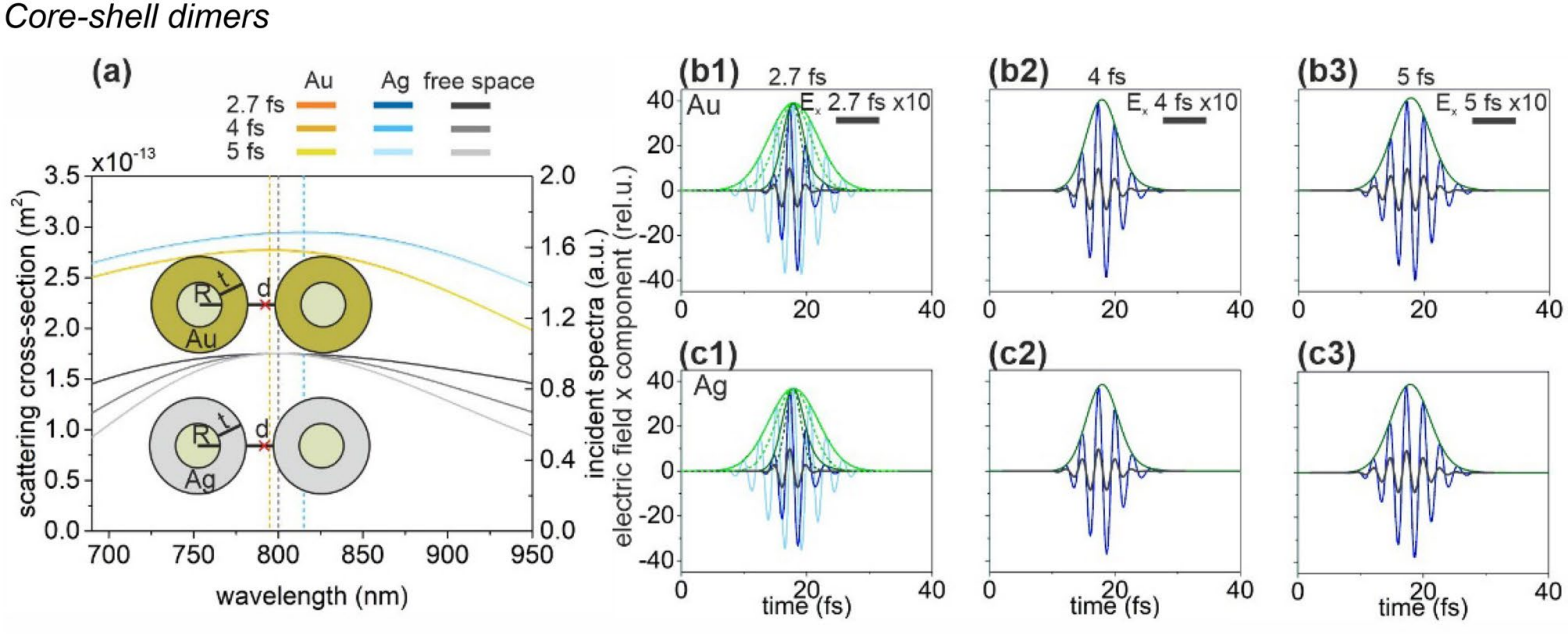
(**a**) Scattering cross-section of optimized silica-metal core–shell dimers and the spectral distribution in free-space in case of illumination by 2.7-fs, 4-fs and 5-fs excitation pulses. Insets: Geometry and optimized parameters of core–shell dimers. Red cross indicates the location of the near-field read-out. (Optimal parameters: R = 32 nm, t = 60 nm, d = 10 nm for silica-Au core–shell dimer and R = 40 nm, t = 55 nm, d = 10 nm for silica-Ag core–shell dimer). Temporal evolution of the enhanced plasmonic field in the gap of (**b**) the silica-gold and (**c**) the silica-silver core–shell dimer; E_x_: 2.7 fs excitation (black), 2.5 NOC criterion (light blue), plasmonic (blue), E_x_ (intensity) envelope: 2.5 NOC criterion solid (dashed) green, plasmonic solid (dashed) olive.

The SCS spectrum of optimized silica-silver and silica-gold core–shell dimers are similar (Fig. 4a). Both the Ag and Au coated dimers are resonant near 800 nm. The SCS peak of the silver-coated core–shell is slightly higher according to its slightly larger size (Fig. 4a). The commensurate FWHM of the silver and gold dimers’ spectrum with the excitation pulses allows a slightly longer exponential decay of the significantly enhanced electric field (Fig. 4b,c, Table 1).

In presence of silver coating, the NOC is slightly lower compared to gold coating for all inspected pulse lengths (Table 1). Due to the ultrabroadband SCS spectrum, the NOC is only slightly higher than that of the exciting pulse. Due to the considerably higher NFE, the ratio NFE/NOC is higher for gold coated core–shell dimers despite the slightly larger NOC for all inspected pulse lengths.

Altogether, the gold-coated core–shell dimer exhibits more advantageous behavior for ultrashort-pulse field enhancement. Changing from core–shell monomers to dimers results in slightly higher NOC both for Au and Ag shells (Table 1). The NFE of Au (Ag) coated core–shell dimer is more than (almost) an order of magnitude higher, than that of the corresponding monomer. The NFE/NOC ratios are significantly higher for dimers as well, representing their advantage over the monomers. Compared to the nanorod dimers, the NFE/NOC ratios are higher (lower) for Au (Ag) core–shell dimers throughout the inspected pulse length intervals, which indicates that the core–shell dimer geometry is better (worse), when the nanoresonator is made of Au (Ag) material.

### Bow-tie nanostructures

**Figure 5:**
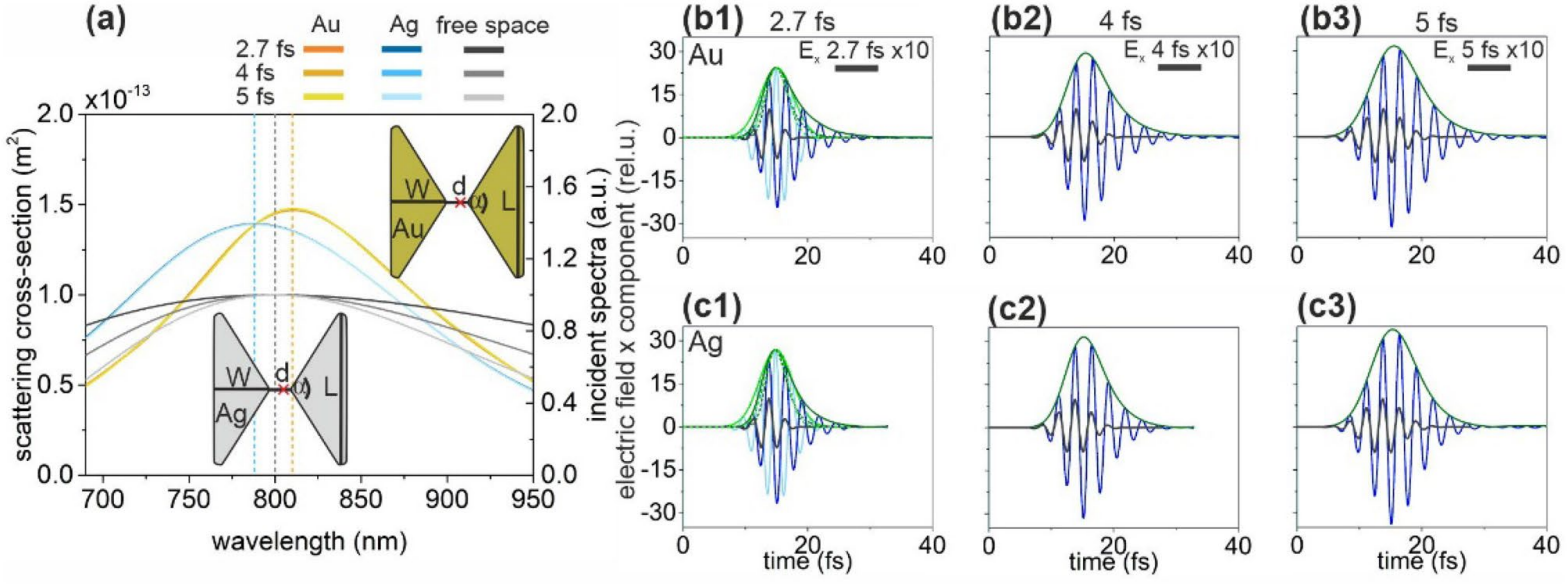
(**a**) Scattering cross-section of optimized bow-ties and the spectral distribution in free-space in case of illumination by 2.7-fs, 4-fs and 5-fs excitation pulses. Insets: Geometry and optimized parameters of bow-ties. Red cross indicates the location of the near-field read-out. (Optimal parameters: L = 160 nm, W = 121 nm, d = 10 nm, t = 49 nm, α = 67° for Au bow-tie and L = 177 nm, W = 121 nm, d = 10 nm, t = 43 nm, α = 72° for Ag bow-tie). Temporal decay of the enhanced plasmonic field in the gap of (**b**) the gold and (**c**) the silver bow-tie; E_x_: 2.7 fs excitation (black), 1.7 NOC criterion (light blue), plasmonic (blue), E_x_ (intensity) envelope: 1.7 NOC criterion solid (dashed) green, plasmonic solid (dashed) olive.

Optimization of bow-tie structures results in a similar geometry and similarly but reversely detuned SCS spectra (Fig. 5a). The SCS resonance peaks are considerably narrower than the spectrum of the incoming 2.7 fs pulse and they are considerably detuned from 800 nm. As a consequence, the decay of the plasmonic field is longer than that of the excitation pulse but it is accompanied by the most significant enhancement in case of bow-ties made of gold and silver (Fig. 5b,c, Table 1).

The NOC is lower for silver bow-tie, which correlates with its broader spectrum (Table 1). A further advantage of the optimized Ag bow-tie is its higher NFE, which enables larger NFE/NOC pulse-length independently.

Bow-ties show higher NFE with respect to nanorod monomers, moreover much higher than core–shell monomers (Table 1). This, together with the observed NOC values enable that the NFE/NOC ratio of bow-ties outperforms the nanorod and core–shell monomers regardless of their material. The significantly higher figure-of-merit indicates that bow-tie structures may be more advantageous for few-cycle plasmonic applications than either nanorod or core–shell monomers.

The bow-ties possess slightly higher NFE/NOC values as compared to nanorod dimers (except Au at 2.7 fs, Table 1). The NFE/NOC is lower (higher) in Au (Ag) bow-ties than that achievable via Au (Ag) core–shell dimers caused by the considerably (significantly) higher NFE and significantly (considerably) higher NOC, respectively.

### Pulse length dependence

**Figure 6:**
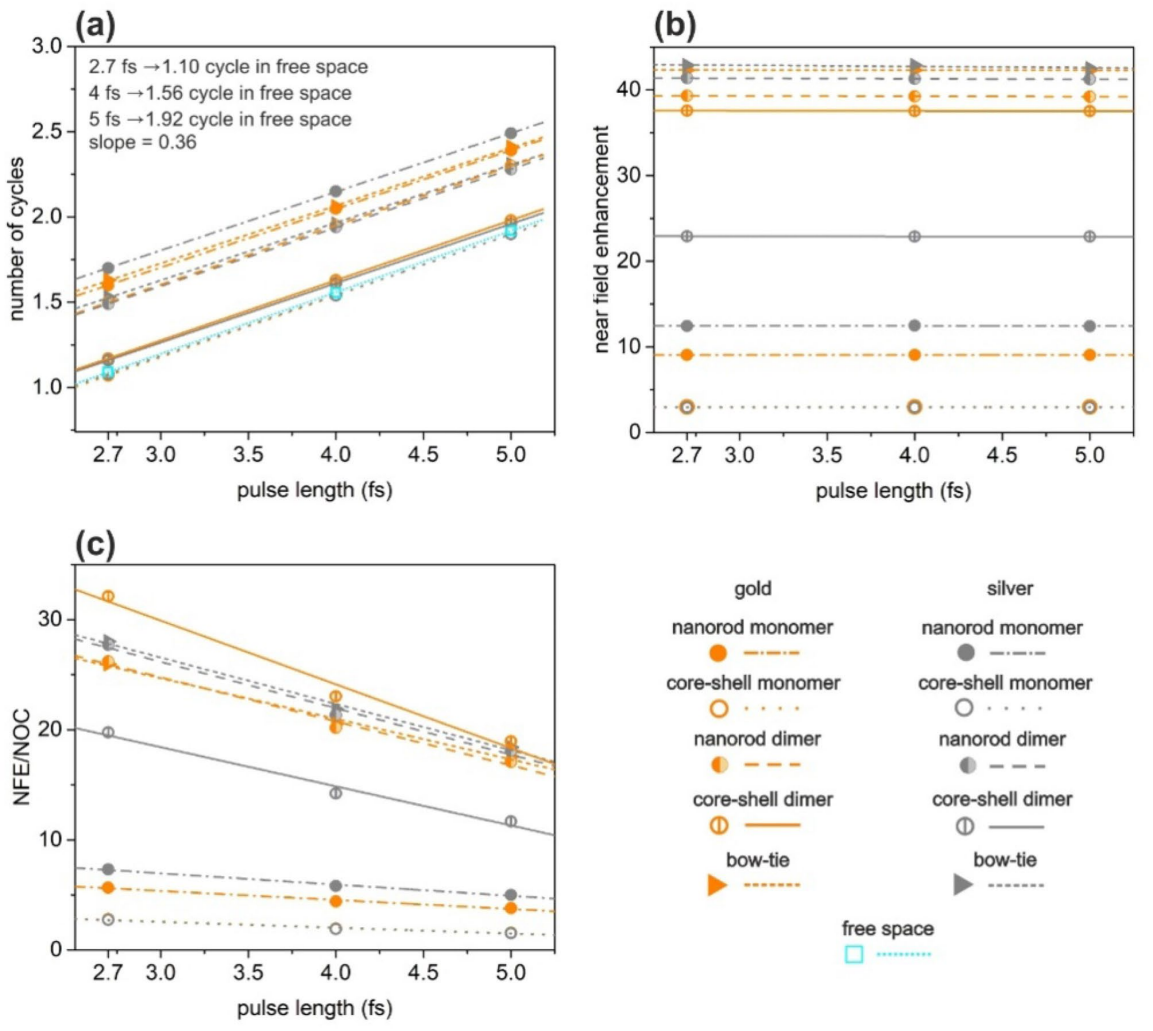
Pulse length dependence of (**a**) number of cycles in the excited plasmonic fields, (**b**) near-field enhancement and (**c**) ratio of the near-field enhancement and number of cycles of the optimized nanostructures. (**a**) also shows the NOC values for a free-space beam for reference.

In Fig. 6, we also present the NOC, NFE and NFE/NOC values as a function of pulse length. Figure 6a shows that in the range of excitation pulse lengths that we have investigated, the NOC of the plasmon field increases directly proportionally with the pulse length (see also in Table 1). Slight deviations are the result of a numerical noise. The rate of increment depends on the nanoresonator type and material as well. The lowest NOC is reachable with Ag and Au coated core–shell monomers for all inspected pulses.

As it is expected, the observed near-field enhancement is independent of the pulse length (Fig. 6b, Table 1). The highest NFE is achievable with Ag bow-ties independently of the pulse length (Fig. 6b). Relatively higher enhancements can be reached with silver nanoresonators in case of nanorod monomers and dimers as well as in case of bow-ties, but gold nanoresonators turned out to be advantageous in case of core–shell monomers and dimers.

As it is evidenced by Fig. 6, the highest NFE/NOC value is achieved with gold core–shell dimers, hence these are the most appropriate nanoresonators for few-cycle near-field enhancement, for all inspected pulse lengths (Fig. 6c, Table 1). Silver bow-tie and silver nanorod dimer exhibits the second and third highest NFE/NOC values, independently of the excitation pulse length.

The relation of slopes between the three different structure types and two materials is complex (Table 1). NOC slopes are always the same for gold and for silver regardless of structure type (except the nanorod dimers, where it is slightly lower for silver).

Although, the NFE/NOC *vs*. incoming pulse-length qualifying a decreasing tendency is lower for Au nanorod monomer, the NFE/NOC remains higher for the Ag nanorod throughout the inspected interval, due to the pulse length independently larger NFE (Table 1). This correlates with the larger ACS, which is achieved despite the larger detuning in case of Ag nanorod monomer. The optimized gold nanorod turns out to be advantageous for longer exciting pulses outside the inspected pulse-length range.

Based on the larger NFE and NFE/NOC Au core–shell monomer is better in the inspected pulse-length interval, which is due to the larger ACS accompanying a resonance near 800 nm. The slightly lower NFE/NOC *vs*. pulse length slope indicates that for longer pulses, the use of silver is more advantageous compared to its gold counterpart in core–shell monomers (Table 1).

Although, ACS (SCS) detuning from the central wavelength is just slightly higher (lower) in case of Au nanorod dimers, the NFE is considerably higher in case of silver nanorod dimers, due to its material properties and due to the smaller size of the components (Table 1). Moreover, gold nanorod dimers exhibit just slightly higher NOC. The silver nanorod dimer is a more reasonable choice based on its considerably higher NFE and slightly more advantageous NOC behavior in case of longer pulses. Accordingly, silver nanorod dimers are better in NFE/NOC independently of the pulse length, however gold keeps becoming better for longer pulses due to its lower NFE/NOC slope.

In contrast, gold core–shell dimer exhibits higher NFE/NOC ratio, as a result of commensurate NOC and considerably higher NFE, which makes it a reasonable choice instead of silver core–shell dimer counterpart for short pulse amplification (Table 1). The considerably higher NFE of the optimized Au coated core–shell dimers correlates with the higher ACS and lower SCS. However, silver keeps becoming better for higher pulse lengths, according to its lower NFE and NFE/NOC slopes.

Change from monomers to dimers is advantageous considering NOC in case of nanorods (Table 1). The NFE achieved via nanorod and core–shell dimers is also considerably higher than that achieved via the corresponding monomers. The NFE/NOC ratios are significantly higher for dimers proving their advantage compared to the monomers. Dominantly monomers may be considered as targets for amplification of longer pulses according to their lower NFE/NOC slopes with respect to dimers both for nanorods and core–shell nanoresonators.

Similarly to nanorod dimers, the silver bow-tie allows to reach smaller NOC and slightly higher NFE, as a result higher NFE/NOC value is achieved (Table 1). Thus, the silver bow-tie is more advantageous than its gold counterpart. This is due to the fact that despite the slightly larger SCS detuning, the NFE is higher for silver bow-tie structures at the same inter-particle distance in accordance with the significantly smaller ACS detuning and with its material properties. However, gold keeps becoming better for longer pulses, according to its lower NFE/NOC slope.

## Conclusions

Comparing all dimers, the Ag bow-tie has the highest NFE. The NOC is the lowest in case of Au and Ag core–shell dimers. Gold core–shell dimers outperform all other dimers in their NFE/NOC ratio due to the relatively low NOC and strong NFE. In case of nanorod dimers and bow-tie structures, the use of silver is most advantageous based on its higher NFE and NFE/NOC ratio.

Comparing all inspected nanoresonators, all types have some advantageous characteristic property. Gold and silver core–shell monomers exhibit the lowest NOC, silver bow-tie and gold core–shell dimer possesses the highest NFE and NFE/NOC values, respectively. Based on our results, we propose Ag bow–tie, Au core–shell and Ag nanorod dimers for the generation of few-cycle plasmonic fields.

Our comparative study shows that the generation of few-cycle plasmonic transients is indeed possible, however this requires optimization of the nanostructures so that best results are achieved. In addition, a trade-off has to be found between the contradictory requirements of large field enhancement and short transients. Depending on the given application, such an optimization can yield satisfactory results. The conditional optimization method that we applied in this study is an important step forward towards adaptive near-field control. These results have the potential to enhance applications in strong-field physics, such as the harmonic conversion of light in the vicinity of nanostructured surfaces. In addition, applications in ultrafast switching and processing of optical signals with surface-integrated nanophotonic structures are also envisaged.
